# Genomic DNA-based measurable residual disease monitoring in pediatric acute myeloid leukemia: unselected consecutive cohort study

**DOI:** 10.1038/s41375-023-02083-9

**Published:** 2023-11-24

**Authors:** Marketa Zaliova, Jan Zuna, Lucie Winkowska, Iveta Janotova, Justina Skorepova, Julius Lukes, Claus Meyer, Rolf Marschalek, Zbynek Novak, Jiri Domansky, Jan Stary, Lucie Sramkova, Jan Trka

**Affiliations:** 1 CLIP (Childhood Leukaemia Investigation Prague), Prague, Czech Republic; 2 Department of Paediatric Haematology and Oncology, Second Faculty of Medicine, Charles University, Prague, Czech Republic; 3 University Hospital Motol, Prague, Czech Republic; 4 Institute of Pharmaceutical Biology/Diagnostic Center of Acute Leukemia (DCAL), Goethe-University, Frankfurt am Main, Germany; 5 Department of Pediatrics, Faculty of Medicine and Dentistry, Palacky University and University Hospital Olomouc, Olomouc, Czech Republic; 6 Pediatric Oncology Department, University Hospital and Faculty of Medicine, Masaryk University, Brno, Czech Republic

**Keywords:** Translational research, Clinical genetics

## Abstract

Measurable residual disease (MRD) monitoring in childhood acute myeloid leukemia (AML) is used to assess response to treatment and for early detection of imminent relapse. In childhood AML, MRD is typically evaluated using flow cytometry, or by quantitative detection of leukemia-specific aberrations at the mRNA level. Both methods, however, have significant limitations. Recently, we demonstrated the feasibility of MRD monitoring in selected subgroups of AML at the genomic DNA (gDNA) level. To evaluate the potential of gDNA-based MRD monitoring across all AML subtypes, we conducted a comprehensive analysis involving 133 consecutively diagnosed children. Integrating next-generation sequencing into the diagnostic process, we identified (presumed) primary genetic aberrations suitable as MRD targets in 97% of patients. We developed patient-specific quantification assays and monitored MRD in 122 children. The gDNA-based MRD monitoring via quantification of primary aberrations with a sensitivity of at least 10^−4^ was possible in 86% of patients; via quantification with sensitivity of 5 × 10^−4^, of secondary aberrations, or at the mRNA level in an additional 8%. Importantly, gDNA-based MRD exhibited independent prognostic value at early time-points in patients stratified to intermediate-/high-risk treatment arms. Our study demonstrates the broad applicability, feasibility, and clinical significance of gDNA-based MRD monitoring in childhood AML.

## Introduction

Acute leukemia is the most common malignant disease in children. Its prognosis has gradually improved over the last decades to today’s ~90% 5 year overall survival (OS) in acute lymphoblastic leukemia (ALL) [1] and ~70% in acute myeloid leukemia (AML) [2], partly due to the adjustment of treatment intensity to the risk of treatment failure, which is now mainly determined by the presence of genetic aberrations and early response to treatment analyzed at the level of measurable residual disease (MRD) [3].

The most commonly used method for MRD detection in current treatment protocols for ALL is quantification of leukemia-specific clonal rearrangements of immunoreceptor genes (IG/TR) at the genomic DNA (gDNA) level by quantitative PCR (qPCR). Various rearrangements of IG/TR are found in the vast majority of ALLs, across different subtypes, and their quantification is well standardized [4]. In AML, IG/TR rearrangements are rare [5] and there is no similar universal genetic MRD target. However, various primary clonal genetic aberrations can be found in virtually all AML cases, and can be used as targets for MRD monitoring. The most common genetic aberrations in pediatric AML are fusion genes, followed by point or small-scale mutations [6]. The success of their identification depends on the extent of genetic testing; the absence of targets in a significant proportion of patients before the use of modern genomic technologies in diagnostics was probably one of the factors that historically favored flow cytometry (FC)-based MRD monitoring over the genetic approaches [7–9].

To date, the most widely used genetic targets for MRD monitoring in pediatric AML have been the *PML::RARA*, *RUNX1::RUNX1T1*, and *CBFB::MYH11* fusion genes [10]. Due to the difficulty of obtaining genomic breakpoint sequences (particularly before the era of modern genomic technologies), quantification of the fusion transcripts has been widely adopted. Although such mRNA-based MRD monitoring is methodologically simple, it does not allow accurate quantification of the amount of residual leukemia cells due to the variable number of transcript copies in individual cells. Moreover, the level of fusion gene expression varies significantly among patients and may limit sensitivity of MRD monitoring [11]. Recently, we have shown that genomic sequences of the three aforementioned fusion genes can be obtained quickly and reliably using next-generation sequencing (NGS) and that gDNA approach enables sensitive MRD monitoring [11]. We compared mRNA- and gDNA-based approaches in 23 patients with *PML::RARA*-/*RUNX1::RUNX1T1*-/*CBFB::MYH11*-positive AML and demonstrated, that mRNA approach may underestimate or (less frequently) overestimate MRD level [11]. A successful detection of the genomic fusion sequences and their use for MRD monitoring has been subsequently demonstrated also by other researchers [12].

Herein, we investigated the applicability and prognostic value of the gDNA-based MRD monitoring in all pediatric AML subtypes in a consecutive, unselected cohort of 133 children. We demonstrate that when using NGS in diagnostics, genetic targets can be found, and gDNA-based MRD monitoring can be used routinely in up to 90% of patients, with the same quality (specificity and sensitivity) as has been performed for many years in pediatric ALL.

## Materials, subjects and methods

### Patients and samples

This study includes 133 out of 135 children (<18 years) that were consecutively diagnosed with primary AML in the Czech Republic between June 2012 and May 2022; diagnostic material was not available in two children, who were excluded from this study.

The majority of children (*n* = 106) were treated according to the AML-BFM 2012 Registry protocol (Supplementary Fig. 1), 14 children with Down syndrome and AML M7 (DS-AMKL) according to the ML-DS 2006 trial protocol (EudraCT trial #2007-006219-2), 10 children with acute prolymphocytic leukemia (APL) according to the amended AML-BFM 2012 Registry protocol, 2 children with APL according to the ICC APL study 02 protocol (NCT04793919) and one child with *FLT3-ITD*-positive *NPM1*-mutated AML was enrolled into CPKC412A2218 trial (NCT03591510). MRD monitoring was not used for risk stratification, but MRD monitoring for research purposes was performed in bone marrow (BM) at time-points defined by treatment protocols and when indicated by the physicians. Peripheral blood (PB) was occasionally analyzed in parallel with BM. In some patients, MRD monitoring continued after treatment either in BM (after hematopoietic stem cell transplantation, HSCT) or in PB. Biological samples were processed and nucleic acids were isolated according to standard laboratory procedures. Diagnostic and treatment procedures, protocols and the research study were approved by the Ethics Committee of University Hospital Motol (NU20-07-00322) and conducted in accordance with the Declaration of Helsinki. Written informed consent was provided by patients or their legal guardians.

### Genetic investigations

The diagnostic algorithm is shown on Supplementary Fig. 2. Routine diagnostics included screening of gene fusions and mutations required for the risk stratification. Additional fusion genes were screened and the *GATA1* gene sequenced in AML M7. Initially, fusions were screened at the mRNA level by in-house developed reverse transcription polymerase chain reaction (RT-PCR) assays and mutations were analyzed at the gDNA level by Sanger sequencing. In later years of the study, these methods were replaced by commercially available quantitative (q) RT-PCR assay (HemaVision®-28Q; DNA Diagnostics A/S, Risskov, Denmark) and targeted NGS. Cases with negative results of fusion gene and mutation screening were analyzed by whole transcriptome sequencing (WTS) either retrospectively (for the purpose of this study) or prospectively (as a part of routine diagnostics).

### Whole transcriptome sequencing

Whole transcriptome sequencing was performed as described previously [13], defuse [14] and Cicero [15] callers were used for fusion and structural variant identification, SNV/indel calling was performed using VarScan and Samtools.

### Mutation screening by targeted NGS

Sequencing libraries were prepared from 200 ng of gDNA using Agilent SureSelect QXT Kit (Agilent Technologies, Santa Clara, California, USA) for the sequencing on NextSeq500 or using SureSelect XT Low input Reagent Kits (Agilent Technologies) for the sequencing on MiSeq instrument (Illumina, San Diego, California, USA). SureSelect Custom designed probes (Agilent Technologies) were used for target enrichment (Supplementary Table 1). NextSeq 500/500 Mid Output v2.5 Kit or MiSeq Reagent Kit v2/v3 (Illumina) were used for the sequencing on NextSeq500 (2 × 150 base pairs) or MiSeq (2 × 250 base pairs), respectively.

### Genomic fusion identification

In a proportion of patients with *KMT2A*-rearranged (*KMT2A*-r) AML, genomic fusions were identified at the Diagnostic Center of Acute Leukemia (DCAL) of Goethe-University using end-point long-distance inverse and/or multiplex PCR followed by Sanger sequencing or NGS as described previously [16, 17]. In remaining patients, genomic fusions were identified by targeted NGS. Libraries were prepared from 50–220 ng of gDNA from diagnostic samples. SureSelect Custom designed probes targeting regions of fusion breakpoints (Supplementary Table 2) were used for the target enrichment. Sequencing was performed as described for mutation screening above.

### Measurable residual disease monitoring by qPCR and qRT-PCR

Gene fusions or mutated allele sequences were used to design primers and probes for qPCR (qRT-PCR) assays. Serial dilutions (10^−1^ to 10^−5^) of patients’ diagnostic gDNA (cDNA) into non-leukemic gDNA (cDNA) prepared from buffy coats of healthy donors were used to create standard curves and to assess quantitative range and detection sensitivity in each PCR run. Experimental set-up, assessment of quantitative range and sensitivity, and results interpretation followed the standards of EuroMRD international network [4]. The *ALB* gene (*GUS* transcript) was used to normalize target level to gDNA (cDNA) input. Quantifiable MRD levels were expressed relative to diagnosis.

### Measurable residual disease monitoring by NGS

Mutation spanning regions (250–350 bp) were amplified from 500 ng of gDNA by single-round PCR using primers composed of a gene specific part and adapter and index sequences (Supplementary Methods). Sequencing with expected output of 10^6^ reads was performed on MiSeq using MiSeq Reagent Kit v2/v3. To determine sensitivity of mutation detection, 10^−4^ and 10^−5^ dilutions of diagnostic gDNA in non-leukemic gDNA prepared from buffy coats of healthy donors were sequenced. Similarly to qPCR, MRD levels were expressed relative to diagnosis.

### Statistical analysis

Differences between MRD levels of two groups were analyzed by Fisher’s exact test. Event-free survival (EFS) and OS were calculated from diagnosis to first failure (death/relapse/secondary malignancy) or to death, respectively. Survival rates were calculated according to Kaplan-Meier and compared by log-rank test. For multivariate analysis, Cox proportional hazards models were constructed for EFS and OS using MRD measured at day 28 or 56, (cyto)genetic risk and treatment arm as tested variables and the model selection was performed using the Akaike Information Criterion (AIC). Results of the best model (based on AIC) are presented.

## Results

### Genetic characterization

For risk stratification and identification of genetic aberrations suitable as MRD targets, diagnostic material from 133 children with primary AML was examined (see “Methods” and Supplementary Table 3). The presence of selected fusion genes and mutations was prospectively investigated using (q)RT-PCR and sequencing. A primary genetic aberration was found in 102 children, while the remaining 31 children were further investigated using WTS. In 20 children, WTS identified rare or novel (presumably) primary genetic aberrations not included in the targeted screening, while seven children were found to possess fusion genes included in the targeted screening but not detected due to atypical variants.

The main genetic findings are summarized in Table 1 (for more details see Supplementary Table 3). A (presumably) primary genetic aberration was found in 97% of cases (129/133), and these AMLs are hereafter referred to as genetically classified. A large proportion of AMLs (81%) were classified into common subtypes: AML with *KMT2A*-r, *PML::RARA*, *RUNX1::RUNX1T1*, *CBFB::MYH11*, mutations (m) of *GATA1*, *CEBPA* or *NPM1*, respectively.

**Table 1:** Genetic and morphological (FAB) classification.

Genetic class		Number of cases	% of total (133)	*FLT3*-ITD^a^	*WT1*m^a^	FAB subtype^b^											
						M0	M1	M1/M2	M2	M2/M4	M3	M4	M4eo	M5	M6	M6/M7	M7
*KMT2A*-r (n=37)	*KMT2A::MLLT3*	16	28%									1		15			
	*KMT2A::MLLT10*	12										3		9			
	*KMT2A::ELL*	3										1		2			
	*KMT2A::AFDN*	2										1		1			
	*KMT2A::SEPT9*	1												1			
	*KMT2A::PRPF19*	1												1			
	*KMT2A::MLLT11*	1												1			
	*KMT2A::ABI1*	1										1					
*GATA1*m		17	13%													1	16
*PML::RARA*		16	12%	7	3						16						
*CEBPA*m		10	8%		1		4		4			2					
*RUNX1::RUNX1T1*		10	8%		1		2		8								
*CBFB::MYH11*		9	7%		2					1		2	6				
*NPM1*m		9	7%	5	1		2		4			3					
*UBTF*		3	2.3%	2	3		1		1			1					
*KAT6A-*r		3	2.3%									2		1			
*HOXA10-*r		3	2.3%														3
*RUNX1*m		2	1.5%	1		2											
*DEK::NUP214*		2	1.5%	2			1					1					
other rare fusions (n=8)	*NUP98::NSD1*	1	0.8%	1			1										
	*CBFA2T3::GLIS2*	1	0.8%		1	1											
	*BCR::ABL1*	1	0.8%					1									
	*SFPQ::ZFP36L2*	1	0.8%									1					
	*ETV6::CTNNB1*	1	0.8%			1											
	*XPO1::TNRC18*	1	0.8%														1
	*FUS::FEV*	1	0.8%				1										
	*ZEB2::RUNX1*	1	0.8%													1	
No primary genetic aberration found		4	3.0%	2	1	1						1		2			
*FAB subtype total*						*5*	*12*	*1*	*17*	*1*	*16*	*20*	*6*	*33*	*0*	*2*	*20*

^a^*FLT3*-ITD was not screened in 12 cases (6 *KMT2A*-r and 6 *GATA1*m AML), *WT1* was not screened in 19 cases (8 *GATA1*m, 7 *KMT2A*-r, 2 *PML::RARA*, 1 *RUNX1::RUNX1T1* and 1*BCR::ABL1* positive AML).

^b^French-American-British morphological classification.

Genetic aberrations that were identified as recurrent but rare in pediatric AML were found in 16 patients: *UBTF*m, *RUNX1*m, *HOXA10* translocation, *KAT6A::CREBBP*, *KAT6A::LEUTX*, *DEK::NUP214*, *BCR::ABL1*, *NUP98::NSD1* and *CBFA2T3::GLIS2*.

In five patients, fusion genes were identified, that have been described so far to occur sporadically (*SPFQ::ZFP36L2* [18], *XPO1::TNRC18* [19]) or not at all in AML (*ETV6::CTNNB1*, *FUS::FEV*, *ZEB2::RUNX1*). These genetic aberrations were assessed as (presumably) primary, based on the occurrence of these fusions (or fusions involving one of the partner genes) in hematological malignancies [20–22].

In the remaining four patients (3%), no primary genetic aberration was found, but only mutations that frequently occur as secondary in AML (*FLT3-ITD*, *WT1*m, *KRAS*m, *NF1*m, *PTPN11*m and *KIT*m; 1–3 mutations per patient) [23]. These four AMLs are hereafter referred to as genetically unclassified.

### Applicability of gDNA-based MRD monitoring

We aimed to establish MRD monitoring at the gDNA level with a sensitivity of at least 10^−4^ (0.01%) in all patients. The preferred method for MRD monitoring was qPCR, while deep amplicon NGS was considered as a second option.

Three children from our cohort died shortly after AML diagnosis, MRD monitoring was thus relevant for 130 patients (“MRD cohort”), of which 126 had genetically classified AML. In 85 of them, the primary genetic aberrations were gene fusions. Targeted NGS (or PCR) was performed to identify genomic fusion sequences and succeeded in 82 of 84 cases examined. Of note, when analyzing WTS data (available in a proportion of patients), in approximately half of the cases the genomic fusion sequence could be found in retained introns of the fusion gene transcripts and targeted NGS was not necessary (data not shown). In all 82 cases with an identified genomic sequence, qPCR systems with the required sensitivity for target detection were implemented. In the two patients in whom genomic fusion was not found, fusion transcripts were used as MRD targets (*NUP98::NSD1*, *ZEB2::RUNX1*). One patient (*SPFQ::ZFP36L2*-positive) was not investigated for the fusion gene DNA sequence, because a TR gene rearrangement was used as a target for MRD monitoring (IG/TR rearrangements were specifically screened in this patients, based on the described occurrence of the *SPFQ::ZFP36L2* fusion in a leukemia of T-cell origin [20]).

In 41/126 children with genetically classified AML, the aberrations available as targets for MRD monitoring were gene mutations (*CEBPA*m, *NPM1*m, *GATA1*m, *UBTF*m, *RUNX1*m) ranging from single base to complex ones. In 29 children, a quantification system with required sensitivity was implemented (in two of them NGS-based because of insufficient qPCR sensitivity). In four children (three with *GATA1*m and one with *CEBPA*m), sensitivity of detections was suboptimal (5 × 10^−4^) but still acceptable for MRD monitoring. In seven children, we were not able to detect primary aberrations (single base *GATA1*m) with sufficient sensitivity, and MRD was not monitored.

Similarly, in a single patient, we were not able to sensitively detect (presumably) primary *RUNX1*m, but the accompanying subclonal *RUNX1*m was used as MRD target with detection sensitivity of at least 10^−4^. This target was lost at AML relapse (Supplementary Fig. 3).

In 3/4 children with genetically unclassified AML, *FLT3-ITD* (*n* = 2) or *WT1*m (*n* = 1) were used as MRD targets (with detection sensitivity of at least 10^−4^), with the awareness of their potential subclonality.

In addition to the four children mentioned above, 1–2 secondary aberrations (*WT1*m, *FLT3-ITD*) were used as additional MRD targets in another six children with genetically classified AML and quantified in parallel with the primary aberrations (Supplementary Fig. 3). In four patients, the levels of all MRD targets correlated well, whereas in two patients the levels of secondary aberrations were consistent with a subclonal origin. These results illustrate the expected pitfalls of using secondary aberrations as targets for MRD monitoring.

Established quantification systems were used to monitor MRD in 122 children (representing 94% of the MRD cohort), in 120 children by qPCR and in two children by NGS. MRD was monitored using (presumably) primary aberrations as targets at the DNA level with a sensitivity of at least 10^−4^, or eventually 5 × 10^−4^, in 112 and four children, respectively (together representing 89% of the MRD cohort). In six children MRD was monitored at the DNA level, but using secondary aberrations as targets, or at the mRNA level with a sensitivity of at least 10^−4^.

### Diverse dynamics of MRD clearance in distinct AML subtypes

The vast majority of patients were treated according to the AML-BFM 2012 Registry protocol, where therapy consisted of 2 induction and 2–3 additional blocks of chemotherapy (CHT); BM for MRD detection was collected after each block (Supplementary Fig. 1). Therapy of children with DS-AMKL consisted of 4 blocks of CHT and the timing of BM sampling was similar; thus, these two groups of patients (106 patients with genetically classified AML in total) were analyzed together. Patients with APL were mostly treated according to different protocol, MRD clearance of APL is thus shown separately in Supplementary Fig. 4. A single patient with *FLT3-ITD*-positive *NPM1*m AML who was also not treated according to the AML-BFM 2012 Registry protocol was also excluded from the analyses described below.

**Fig. 1:**
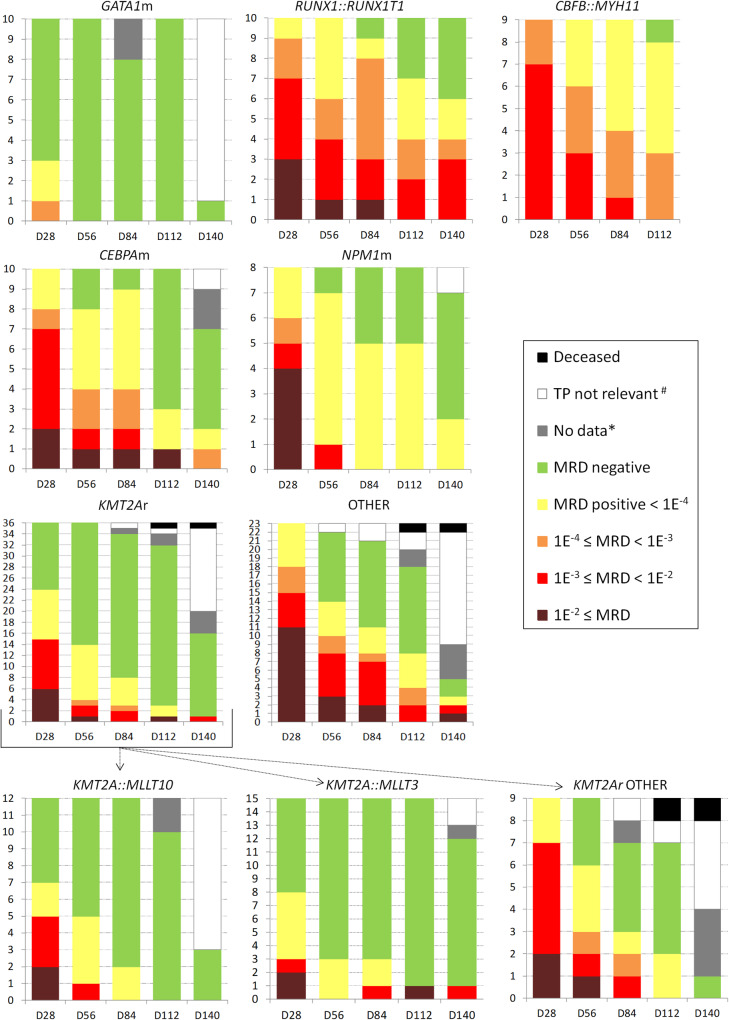
Dynamics of MRD clearance. The figure shows the dynamics of MRD clearance in patients with six different genetic subtypes of AML and patients with other AML subtypes (*BCR::ABL1*, *CBF2A::GLIS2*, *DEK::NUP214*, *ETV6::CTNNB1*, *FUS::FEV*, *HOXA10-*r, *KAT6A-*r, *NUP98::NSD1*, *RUNX1*m, *SFPQ::ZFP36L2*, *UBTF*m, *XPO1::TNRC18*, *ZEB2::RUNX1*) grouped together (OTHER). In AML with *KMT2A*-r, the dynamics of MRD clearance in three subgroups stratified by fusion partner genes is also shown. The *Y*-axis shows patient numbers, the *X*-axis shows treatment time points. ^#^CHT was not administered either because it was not included in the respective treatment arm’s regimen or the patient relapsed/received modified therapy; *BM sampling was not performed or the time point was not reached.

Of all AML subtypes, patients with *GATA1*m AML had the fastest MRD clearance, 70% achieved molecular remission (mREM) after the first CHT block (at day 28; D28) (Fig. 1). Patients with prognostically favorable genetic subtypes (*CBFB::MYH11*, *RUNX1::RUNX1T1*, *CEBPA*m and *NPM1*m) were treated predominantly on the SR arm of the AML-BFM 2012 Registry protocol. Compared with *GATA1*m, their MRD clearance was significantly slower, none achieved mREM at D28 (70% vs. 0%, *p* < 0.0001), most patients (63–78% within individual subtypes) had MRD ≥ 10^−3^ at D28, and a significant proportion of patients (25–89% within individual subtypes) did not achieve mREM after the last CHT block. Patients with *KMT2A*-r AML and AML classified into remaining subtypes were treated predominantly on the intermediate- and high-risk (IR, HR) arms. In the subgroup with *KMT2A*-r, 33% (61%) achieved mREM at D28 (D56), thus their response to treatment was overall faster compared to the four prognostically favorable subtypes listed above (mREM 33% vs. 0% at D28, *p* < 0.0001). Importantly, except for *CBFB::MYH11* AML, initial treatment (up to D56) on the SR, IR and HR arms was identical. There were no significant differences in MRD dynamics between patients with the two most common *KMT2A*-r (*KMT2A::MLLT10* and *KMT2A::MLLT3) *while patients with other *KMT2A*-r had significantly slower MRD clearance (mREM at D28 44% in *KMT2A::MLLT10*/*MLLT3* vs. 0% in other *KMT2A*-r, *p* = 0.02). Remaining patients, treated on the IR and HR arms, had various AML subtypes individually represented only in small numbers; when analyzed together as one genetically heterogeneous group, their response to treatment was worse compared to *KMT2A*-r AML, no patient achieved mREM at D28 (0% vs. 33%, *p* = 0.0018).

### Detection of molecular relapse by MRD monitoring

**Fig. 2:**
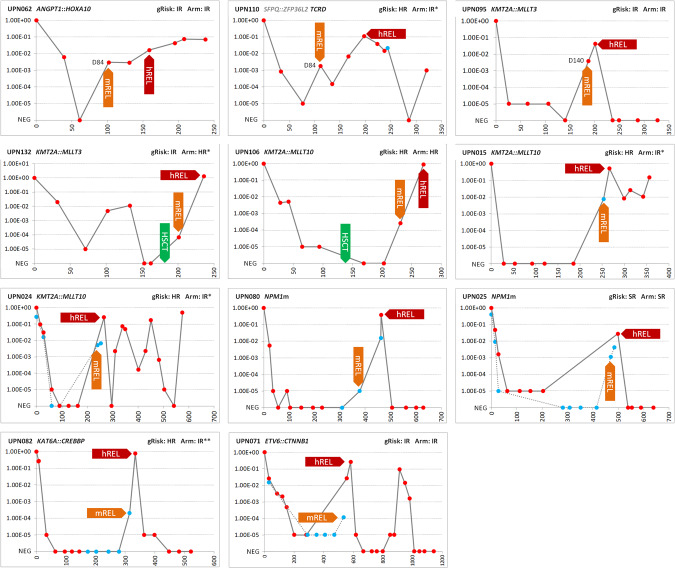
Detection of molecular relapse. The figure shows the course of MRD in 11 patients with detected molecular relapse. The unique numbers and primary aberrations of the patients are shown in the headers of the graphs. Primary aberrations were used as MRD targets in 10 patients, while the *TCRD* gene rearrangement was used as a target in patient UPN110. Three patients who relapsed while still on treatment are shown in the top row. Red circles correspond to BM samples, blue circles to PB samples. The *Y*-axis shows MRD levels, for graphical representation, non-quantifiable positive samples were assigned an MRD value of 1.00E−05. *X*-axis shows time since diagnosis (time 0) in days. gRisk, (cyto)genetic risk (see Supplementary Fig. 1 for risk stratification); Arm, treatment arm of AML-BFM 2012 registry protocol; *patients were treated in a different treatment arm than would correspond to gRISK based on clinicians’ decision; **gRISK was assigned retrospectively based on the corrected cytogenetic result reporting a complex karyotype, treatment followed the originally assigned IR gRISK; mREL molecular relapse, hREL hematological relapse, NEG negative, D day. None of the patients had *WT1*m; *FLT3*-ITD was present only in patient UPN080, who was enrolled in CPKC412A2218 trial and received FLT3-inhibitor.

In order to detect early molecular relapse (mREL), MRD was monitored after treatment in a proportion of patients. To better understand the relevance of MRD measured in PB, together with some BM samples collected at different time-points during and after treatment, PB samples were also collected. The analysis confirmed published observations [24, 25] that MRD levels in PB may be (but not always are) lower compared to BM (Supplementary Fig. 5). Molecular relapse, defined as a reversal of negative MRD to positive or a 1-log increase in MRD positivity confirmed in subsequent sample, was observed in 11 patients (Fig. 2). In five patients, mREL as MRD 10^−5^ to 10^−3^ was detected in BM either during intensive treatment (*n* = 3) or after HSCT (*n* = 2). In six patients, mREL was detected in PB during post-treatment follow-up, in three of them as MRD ≥ 10^−3^. Hematological relapse followed in 13–86 days.

### Prognostic value of MRD monitoring at the gDNA level

**Fig. 3:**
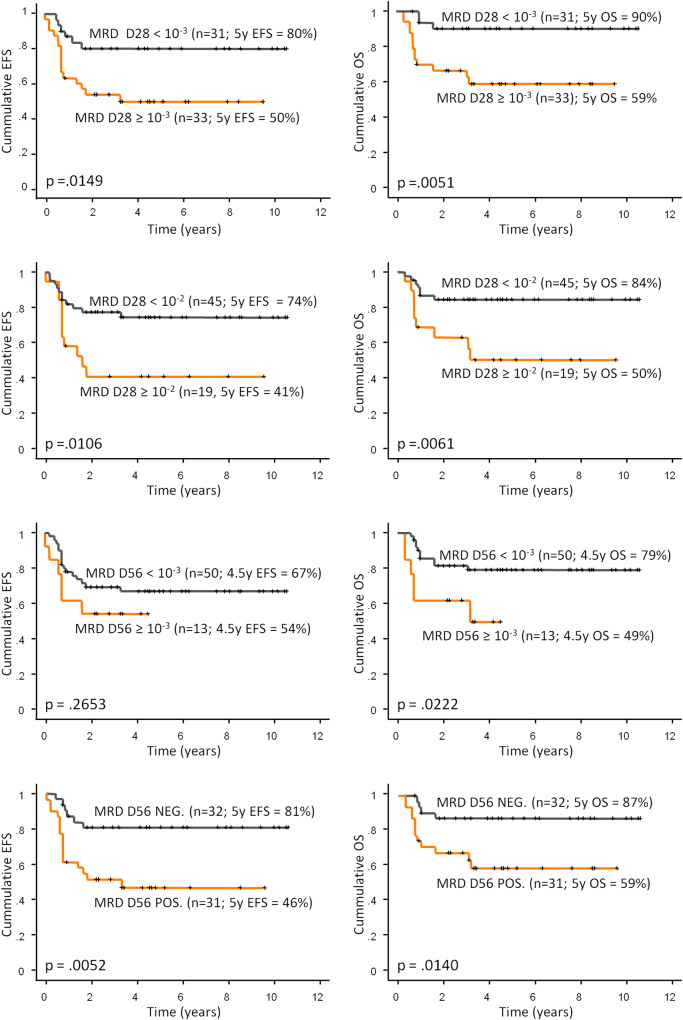
Treatment outcomes of patients stratified by MRD levels. The figure shows treatment outcomes for 64 patients treated in the IR and HR arms of the 2012 BFM AML Registry protocol stratified by MRD levels after 1st induction (D28) or 2nd induction (D56). A total of 68 patients were treated in the IR (*n* = 36) and HR (*n* = 32) arms, MRD was not measured in two of these patients, and the other two patients died before D28. Data on D56 MRD were missing in one patient (BM aspiration was not performed). Of the 64 patients included in the analyses, 35 patients had *KMT2A*-r AML, 26 patients had AML classified into one of the other 17 subtypes (1–3 patients per subtype), and 3 patients had unclassified AML. EFS event-free survival, OS overall survival, y year, NEG. negative, POS. positive. Censoring is indicated by crosses.

We investigated the prognostic value of gDNA-based MRD at early time-points of treatment. Given the excellent treatment outcomes of patients with APL, DS-AMKL, and patients treated on the SR arm of the AML-BFM 2012 registry protocol (*n* = 64, 5-years EFS 97%, median follow-up 3.2 years), we focused on patients treated on the IR and HR arms (*n* = 68, 5 years EFS 62%). This cohort involved nine patients who were reassigned from SR (*n* = 1) or IR (*n* = 8) to HR arm based on poor therapy response (see Supplementary Methods). Significantly different EFS and OS were observed between patients stratified by MRD levels 10^−3^ as well as 10^−2^ at D28 (Fig. 3). Patients stratified by MRD level 10^−3^ at D56 had significantly different OS only, while both EFS and OS were significantly different when patients were stratified by any positivity vs. negativity (Fig. 3). Neither (cyto)genetic risk nor treatment (IR vs. HR arm) had significant prognostic impact (Supplementary Fig. 6). In a multivariate analysis including (cyto)genetic risk and treatment, D28 MRD was the only significant predictor of outcome using both the 10^−3^ (*p* = 0.006 for EFS and 0.012 for OS) and 10^−2^ levels (*p* = 0.004 for EFS and 0.01 for OS) for stratification (Supplementary Table 4). In the same model but with D56 MRD, MRD was again the only significant predictor of outcome, whereas stratification by any positivity vs. negativity had stronger predictive value than stratification at 10^−3^ level (positivity vs. negativity: *p* = 0.004 for EFS and *p* = 0.022 for OS; 10^−3^ cut off: no significant difference for EFS, *p* = 0.029 for OS).

## Discussion

The main objective of our study was to evaluate the feasibility, potential pitfalls and clinical relevance of the gDNA-based MRD monitoring in pediatric AML. The prerequisite for this MRD monitoring is the identification of genetic targets, representing clonal genetic aberrations of leukemic cells. To cover the full spectrum of potential aberrations, ranging from point mutations to fusion genes, we used a two-step algorithm of genetic investigations comprising targeted screening and WTS (the algorithm was adopted and is now used as a routine diagnostic procedure). A (presumably) primary (i.e., clonal) genetic aberration representing a potential MRD target was found in 97% of patients, where in 20% of patients this succeeded only thanks to WTS (which can detect both mutations and fusion genes). Since fusion genes are usually screened at the mRNA level, the corresponding gDNA sequence must be subsequently identified to be used for gDNA-based MRD monitoring. The success rate of targeted NGS in identifying genomic fusions was nearly 100% in our cohort. As a targeted method, it covers a fixed spectrum of aberrations and is therefore not applicable to patients with rare fusions outside this spectrum. However, we found that in approximately 50% of cases the gDNA sequence of fusions can be identified using WTS, used for the rare fusions detection. Overall, our data on an unselected cohort show that the absence of MRD target is very rare when utilizing NGS in diagnostics.

The applicability of identified clonal aberrations for the gDNA-based MRD monitoring with a sensitivity of at least 10^−4^ was 86% (112/130), and with a sensitivity of at least 5 × 10^−4^ 89% (116/130). Almost all single-base mutations, which occurred mainly in the *GATA1* gene, turned out to be inapplicable for a sensitive MRD monitoring in our hands. For target quantification, we preferentially used patient-specific qPCR, which has a major advantage over NGS in that the rules for implementation and interpretation are generally accepted by the expert community (originally developed for immunoreceptor gene rearrangements in ALL) [4]. These rules address, among other things, how to deal with the determination of sensitivity in the presence of background nonspecific amplification. Since such rules are not yet fully established for NGS, we used NGS only for large mutations with no sequencing background.

The clonal origin of the aberration (its presence in all leukemic cells) is important for its use in MRD detection, as subclonality can significantly bias the actual MRD values. We attributed the primary origin to aberrations based on their nature and available data on recurrence, mutual exclusivity with other aberrations, and persistence in relapse. However, we cannot rule out the possibility that some of the aberrations considered by us to be primary were in fact subclonal. Such risk is virtually unavoidable and applies (even more significantly) also to the well-established IG/TR-based MRD monitoring in ALL. In seven patients, we deliberately used as MRD targets aberrations whose subclonal origin was clear or probable (but with allelic frequency that indicated their presence in most leukemia cells). Subclonality led to the underestimation of MRD and/or loss of target at relapse in three cases. Nevertheless, we believe that in patients with no primary aberration found, MRD monitoring using (potentially) subclonal aberrations as targets may still be at least partially beneficial. Due to the risk of MRD underestimation, a low MRD level is not a reliable indicator of good response to treatment; in contrast, a high MRD level might be a good enough indicator of poor response to treatment even with a subclonal target.

The most important reason for MRD monitoring is to assess early response to treatment for the risk-stratification purposes. Consistent with previously published data, we have shown that MRD clearance dynamics vary substantially between AML subtypes and that specific MRD levels at different time points do not have the same prognostic significance in all subtypes [25, 26]. Paradoxically, markedly slow MRD clearance, which does not correlate with treatment outcome, occurs in prognostically favorable subtypes. On the other hand, our data demonstrate that in patients with less favorable subtypes, slow clearance of the gDNA-based MRD has strong prognostic significance independent of (cyto)genetic risk and treatment.

Post-treatment MRD can also be used for early detection of imminent relapse. In line with previous studies, our data show that the development of relapse can be rapid without much room for therapeutic intervention before progression to hematological relapse [27, 28]. Therefore, frequent sampling and deep sensitivity of MRD monitoring are essential. Some previous studies have suggested that due to high expression of fusion genes or mutated alleles, the mRNA-based MRD may be more sensitive than gDNA-based MRD [29]. However, such data are lacking for AML subtypes with higher relapse rates, and therefore we are not convinced of the benefit of using mRNA-based MRD even for this purpose.

Prognostic value of MRD in early treatment has been demonstrated previously, mainly by studies using the FC-based MRD detection [30–35]. Existing MRD-based treatment stratifications in pediatric AML thus mostly rely on this method. However, it has to deal with lower specificity of leukemia-associated phenotype, immunophenotypic heterogeneity of the myeloid leukemic populations and changes of immunophenotype during treatment. A recent study suggested, that FC-based MRD monitoring is less sensitive compared to the gDNA-based approach and may provide both false negative and false positive results [12].

Our gDNA-based approach was applicable in a standardizable manner to 90% of an unselected cohort of patients across all AML subtypes. It is now routinely used in our laboratory for MRD monitoring in children diagnosed with AML in the Czech Republic. The laboriousness of the procedure is not significantly different from the quantification of IG/TR rearrangements in ALL (including the use of NGS for target identification), which has been widely standardized and is used universally in real-life ALL treatment already for two decades.

In summary, we present a strategy for MRD monitoring in pediatric AML that is technologically feasible, applicable to the vast majority of all patients, and has clear prognostic significance.

### Supplementary information

Supplemental Appendix

Supplementary Table 3. Patients’ characteristics

## Data Availability

The data that support the findings of this study are available from the corresponding author upon reasonable request.
