# Supplementary material for: Genomic DNA-based measurable residual disease monitoring in pediatric acute myeloid leukemia: unselected consecutive cohort study
Source: Leukemia. 2023 Nov 24;38(1):21–30. doi: 10.1038/s41375-023-02083-9 (PMC10776399; doi:10.1038/s41375-023-02083-9)
Supplement: Supplementary file 1 — Supplemental Appendix [file 41375_2023_2083_MOESM1_ESM.pdf]

Zaliova et al.: Genomic DNA-based measurable residual disease monitoring in pediatric acute myeloid leukemia: unselected consecutive cohort study

SUPPLEMENTAL APPENDIX

Supplemental Methods

AML-BFM 2012 Registry protocol

According to the AML-BFM 2012 registry protocol patients were stratified into 3 risk groups based on (cyto)genetics and early response to treatment (assessed by morphology or flow cytometry). Patients with *PML::RARA*, *RUNX1::RUNX1T1*, *CBFB::MYH11*, *KMT2Ar::MLLT11* and patients with *CEBPA* or *NPM1* mutation with normal karyotype were assigned to the standard risk (SR) arm. Patients with *KMT2A::MLLT10/AFF1/AFDN*, *DEK214:NUP98*, *NSD1::NUP98*, *ETV6::MNX1*, *BCR::ABL1*, *WT1* mutation combined with *FLT3*-ITD, complex karyotype (and without favorable aberration and without *KMT2A* rearrangements), 12p aberrations, t(2;22) or monosomy 7 (and without favorable aberration) were assigned to the high risk (HR) arm. Patients with other (cyto)genetic findings than the above listed ones were assigned to the intermediate risk (IR) arm. Patients assigned to the SR (IR) arm based on (cyto)genetics but with ≥10% blasts in bone marrow after the 1st or ≥5% after the 2nd induction (evaluated by morphology) were reassigned to the IR (HR) arm. Patients received 4-5 blocks of intensive combination chemotherapy, which was followed by hematopoietic stem cells transplantation in the HR arm. Out of 106 children, 38 were treated on SR, 35 on IR and 33 on HR arms of AML-BFM 2012 registry. In total, 14 children were reassigned to a different treatment arm than defined by (cyto)genetic risk: 5 patients were reassigned from IR to HR based on morphologically assessed treatment response, 3 from IR and 1 from SR to HR based on clinicians' decisions directed by measurable residual disease (MRD) monitoring, and 5 from HR to IR based on clinicians' decisions to reduce therapy toxicity.

According to the modified 2012 AML-BFM registry protocol, all children with APL received 7 blocks of ATRA and 4 blocks of arsenic trioxide, and those with an initial white blood cell count ≥ 10 000 /µl also received 1 block of chemotherapy (ADx) as a part of induction.

Primer design for MRD monitoring by deep-amplicon NGS

Example primer pair for library preparation (Adapter 1 – ***<u>Index</u>*** – Adapter 2 – **<u>Gene specific part</u>**):

Forward primer

CAAGCAGAAGACGGCATACGAGAT***<u>ACCACTGT</u>***GTGACTGGAGTTCAGACGTGTGCTCTTCCGATCT**<u>CAGCCCGGTCACCCCACG</u>**

Reverse primer

AATGATACGGCGACCACCGAGATCTACAC***<u>CTAGAACA</u>***ACACTCTTTCCCTACACGACGCTCTTCCGATCT**<u>GCTGTTCCACCCGCTTGCG</u>**

Supplemental Table 1. Mutation screening by NGS - gene regions targeted by custom designed probes

Genomic coordinates (hg19)	Gene
chr5:170814902-170815060	*NPM1*
chr5:170817004-170817184	*NPM1*
chr5:170818258-170818478	*NPM1*
chr5:170818659-170818853	*NPM1*
chr5:170819663-170820032	*NPM1*
chr5:170827106-170827264	*NPM1*
chr5:170827792-170827979	*NPM1*
chr5:170832255-170832457	*NPM1*
chr5:170833350-170833459	*NPM1*
chr5:170834653-170834828	*NPM1*
chr5:170837480-170837619	*NPM1*
chr11:32410553-32410775	*WT1*
chr11:32413467-32413660	*WT1*
chr11:32414161-32414351	*WT1*
chr11:32417752-32418003	*WT1*
chr11:32421443-32421640	*WT1*
chr11:32437985-32438136	*WT1*
chr11:32439072-32439250	*WT1*
chr11:32449451-32449654	*WT1*
chr11:32449992-32450215	*WT1*
chr11:32452025-32452135	*WT1*
chr11:32456195-32456956	*WT1*
chr13:28578138-28578361	*FLT3*
chr13:28588538-28588744	*FLT3*
chr13:28589243-28589443	*FLT3*
chr13:28589676-28589888	*FLT3*
chr13:28592553-28592776	*FLT3*
chr13:28597436-28597664	*FLT3*
chr13:28598947-28599130	*FLT3*
chr13:28601174-28601428	*FLT3*
chr13:28602264-28602475	*FLT3*
chr13:28607973-28608594	*FLT3*
chr13:28609581-28609860	*FLT3*
chr13:28610021-28610230	*FLT3*
chr13:28611271-28611475	*FLT3*
chr13:28622361-28622630	*FLT3*
chr13:28623470-28623961	*FLT3*
chr13:28624181-28624409	*FLT3*
chr13:28626631-28626861	*FLT3*
chr13:28631433-28631649	*FLT3*
chr13:28635953-28636256	*FLT3*
chr13:28644577-28644799	*FLT3*
chr13:28674554-28674697	*FLT3*

Genomic coordinates (hg19)	Gene
chr17:42282359-42298722	*UBTF*
chr19:33792193-33793475	*CEBPA*
chr21:36164381-36164957	*RUNX1*
chr21:36171547-36171809	*RUNX1*
chr21:36193914-36194043	*RUNX1*
chr21:36206656-36206948	*RUNX1*
chr21:36231720-36231925	*RUNX1*
chr21:36252803-36253060	*RUNX1*
chr21:36259089-36259459	*RUNX1*
chr21:36265171-36265310	*RUNX1*
chr21:36421088-36421246	*RUNX1*
chrX:48649466-48649786	*GATA1*
chrX:48650200-48650678	*GATA1*
chrX:48650679-48650925	*GATA1*
chrX:48651528-48651754	*GATA1*
chrX:48652149-48652621	*GATA1*

Supplemental Table 2. Genomic fusion identification by NGS - gene regions targeted by custom designed probes

Genomic coordinates (hg19)	Gene
chr1:110881945-110889303	*RBM15*
chr2:61704500-61708400	*XPO1*
chr2:145274000-145279000	*ZEB2*
chr3:25469000-25503000	*RARB*
chr6:18226405-18236831	*DEK*
chr7:27190000-27247000	*HOXA7/9/10/11/13*
chr8:41792104-41800500	*KAT6A*
chr8:93029454-93088280	*RUNX1T1*
chr9:133575020-133730483	*ABL1*
chr9:134025999-134035000	*NUP214*
chr10:76780500-76792000	*KAT6B*
chr11:3740000-3790500	*NUP98*
chr11:85668218-85692271	*PICALM*
chr11:118350000-118373500	*KMT2A*
chr12:11802000-12046000	*ETV6*
chr12:12006000-12037500	*ETV6*
chr12:53609000-53627000	*RARG*
chr15:74315168-74317268	*PML*
chr15:74325497-74326871	*PML*
chr16:15814008-15826565	*MYH11*
chr16:31191431-31206192	*FUS*
chr16:67100585-67132682	*CBFB*
chr16:88943335-88948000	*CBFA2T3*
chr17:38487470-38504716	*RARA*
chr17:46684000-46706000	*HOXB7/8/9*
chr21:36206898-36231771	*RUNX1*
chr22:23523148-23596167	*BCR*
chr22:23631704-23634825	*BCR*
chr22:23653884-23655208	*BCR*

Supplementary Table 3. Patients' characteristics

Supplementary Table 3 with patient data including their AML genetics, risk stratification, treatment, MRD monitoring and outcome is provided as a separate Excel spreadsheet with footnotes.

Supplemental Table 4. Outcome - multivariate analysis

Definition of variables	
(cyto)genetik risk	Three categories - SR, IR and HR as defined by cytogenetic and molecular genetic findings as described in Supplemental Figure 1
treatment	Two categories - IR and HR treatmnet arm as defined in Supplemental Figure 1
D28 MRD	Two categories - version 1: patients with MRD ≥ 10^{-3} and patients with MRD < 10^{-3}, version 2: patients with MRD ≥ 10^{-2} and patients with MRD < 10^{-2}
D56 MRD	Two categories - version 1: patients with MRD ≥ 10^{-3} and patients with MRD < 10^{-3}, version 2: patients with any detectable MRD (positive MRD) and patients with no detectable MRD (negative MRD)

Results of Cox proportional hazard model* for event free survival				
Tested variables: (cyto)genetic risk + treatment + D28 MRD with 10^{-3} cut-off				
	Hazard ratio	lower 95% CI	upper 95% CI	P
treatment - IR arm	2.23	0.93	5.39	0.074
**D28 MRD - < 10^{-3}**	**0.26**	**0.10**	**0.68**	**0.006**
Tested variables: (cyto)genetic risk + treatment + D28 MRD with 10^{-2} cut-off				
	Hazard ratio	lower 95% CI	upper 95% CI	P
treatment - IR arm	2.22	0.90	5.45	0.083
**D28 MRD - < 10^{-2}**	**0.27**	**0.11**	**0.65**	**0.004**
Tested variables: (cyto)genetic risk + treatment + D56 MRD with 10^{-3} cut-off				
null model				
Tested variables: (cyto)genetic risk + treatment + D56 MRD with "any positivity" cut-off				
	Hazard ratio	lower 95% CI	upper 95% CI	P
treatment - IR arm	1.96	0.82	4.65	0.130
**D56 MRD - positive MRD**	**4.14**	**1.59**	**10.79**	**0.004**

Results of Cox proportional hazard model* for overall survival				
Tested variables: (cyto)genetic risk + treatment + D28 MRD with 10^{-3} cut-off				
	Hazard ratio	lower 95% CI	upper 95% CI	P
**D28 MRD - < 10^{-3}**	**0.20**	**0.06**	**0.70**	**0.012**
Tested variables: (cyto)genetic risk + treatment + D28 MRD with 10^{-2} cut-off				
	Hazard ratio	lower 95% CI	upper 95% CI	P
**D28 MRD - < 10^{-2}**	**0.27**	**0.10**	**0.73**	**0.010**
Tested variables: (cyto)genetic risk + treatment + D56 MRD with 10^{-3} cut-off				
	Hazard ratio	lower 95% CI	upper 95% CI	P
**D28 MRD - < 10^{-3}**	**0.32**	**0.12**	**0.89**	**0.029**
Tested variables: (cyto)genetic risk + treatment + D56 MRD with "any positivity" cut-off				
	Hazard ratio	lower 95% CI	upper 95% CI	P
**D56 MRD - positive MRD**	**3.76**	**1.21**	**11.67**	**0.022**

* best model based on Akaike Information Criterion; CI, confidence interval; P, p-value

Supplemental Figure 1. Risk stratification and therapy scheme of AML-BFM 2012 Registry protocol

	I1	D28 ↓ I2	D56 ↓ C3	D84 ↓ C4	D112 ↓ C5/HSCT (D140 ↓)
**SR** (*PML::RARA* [1] a *CBFB::MYH11*)	ADxE	AI	haM	HA(E)	
**SR** (*RUNX1::RUNX1T1* , *NPM1* m/*CEBPA* dm and normal karyotype, *KMT2A::MLLT11*) (d28 ≥ 10% blasts and/or d56 ≥ 5% blasts in BM shifts patients to IR)	ADxE	HAM	AI	haM	HAE
**IR** (d28 ≥ 10% and/or d56 ≥ 5% blasts i BM shifts patients to HR)	ADxE	HAM	AI/2-CDA	haM	HAE
**HR** (*KMT2A::MLLT10* /*AFDN* /*AFF1* , *NUP98::NSD1* , *DEK::NUP214* , *ETV6::MNX1* , *BCR::ABL1* [2], *WT1* m+*FLT3* -ITD, complex karyotype (and no favorable aberrations + no *KMT2A* r), 12p aberrations, t(2;22), monosomy 7 (and without favorable	ADxE	HAM	AI/2-CDA<br>DNX-FLAG[3]	haM<br>FLAG[3]	HSCT

I - induction, C - course; D - day; SR/IR/HR - standard risk/intermediate risk/high risk; m - mutation; dm - double mutation; [1] plus ATRA, [2] plus TKI, [3] DNX-FLAG and FLAG were administered as C3 and C4 only in HR patients with non-response on D56

Supplemental Figure 2. Algorithm for routine molecular-genetic diagnostics

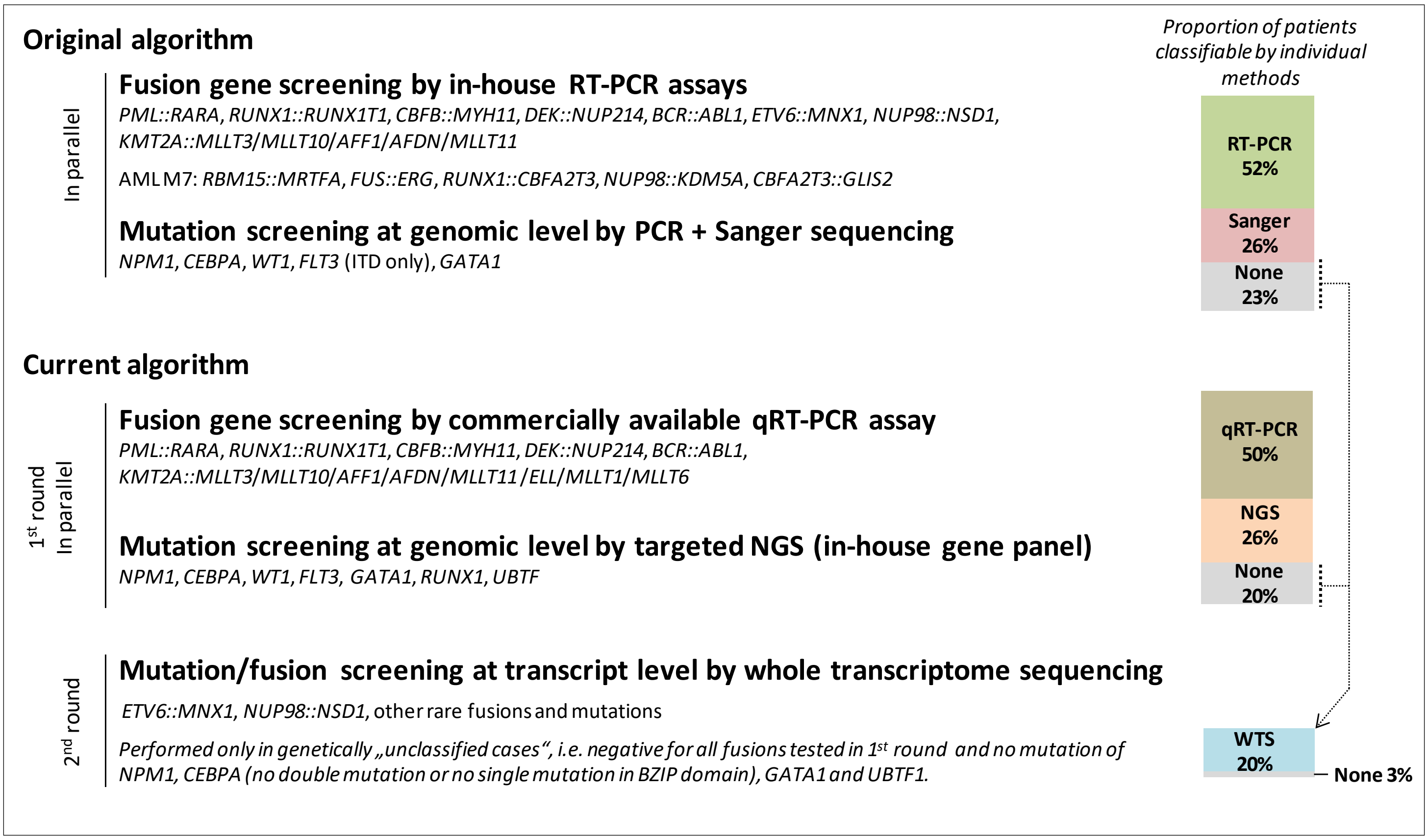

Supplemental Figure 3. MRD monitoring using (potentially) subclonal genetic aberrations as targets

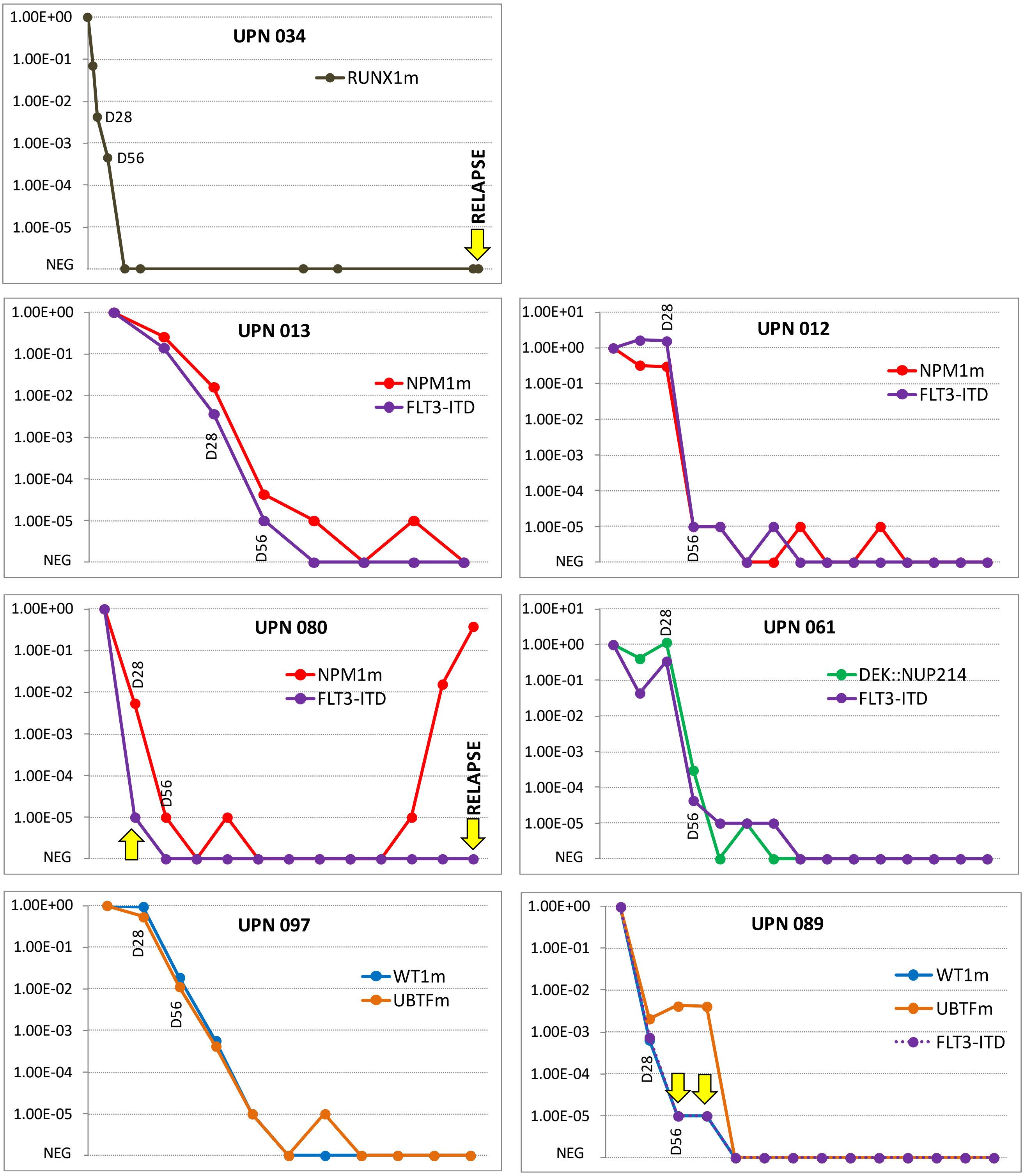

The figure shows the course of MRD in patients (presumed) subclonal aberration used as the sole or additional target for MRD monitoring. Unique patient numbers are listed in the chart headers. For graphical representation, non-quantifiable positive samples were assigned an MRD value of 1.00E-05. Yellow arrows indicate time points when quantification of subclonal aberration underestimated MRD or was a false negative due to target loss. The mutation frequency (MAF) of the *RUNX 1* allele c.343_344insGG, which was used as the MRD target in patient UPN 034, was 17% in the BM diagnostic sample. Two additional *RUNX1*m mutations were found in this patient, a nonsense mutation c.601C>T with a MAF of 41%, which was considered (putatively) primary but could not be sensitively quantified, and another subclonal mutation c.602G>A with a MAF of 12%. In patients UPN080 and UPN089, diagnostic *FLT3*-ITD and *WT1*m levels corresponded to subclonal origin. The diagnostic allelic ratios of *FLT3*-ITD to wild-type *FLT3* were 1.4 and 0.6 in patients UPN080 and UPN089, respectively, and the MAF of *WT1*m was 39% in patient UPN089. Patient UPN080 was treated by FLT3-inhibitor. D, day; NEG, negative.

Supplemental Figure 4. MRD clearance in patients with APL

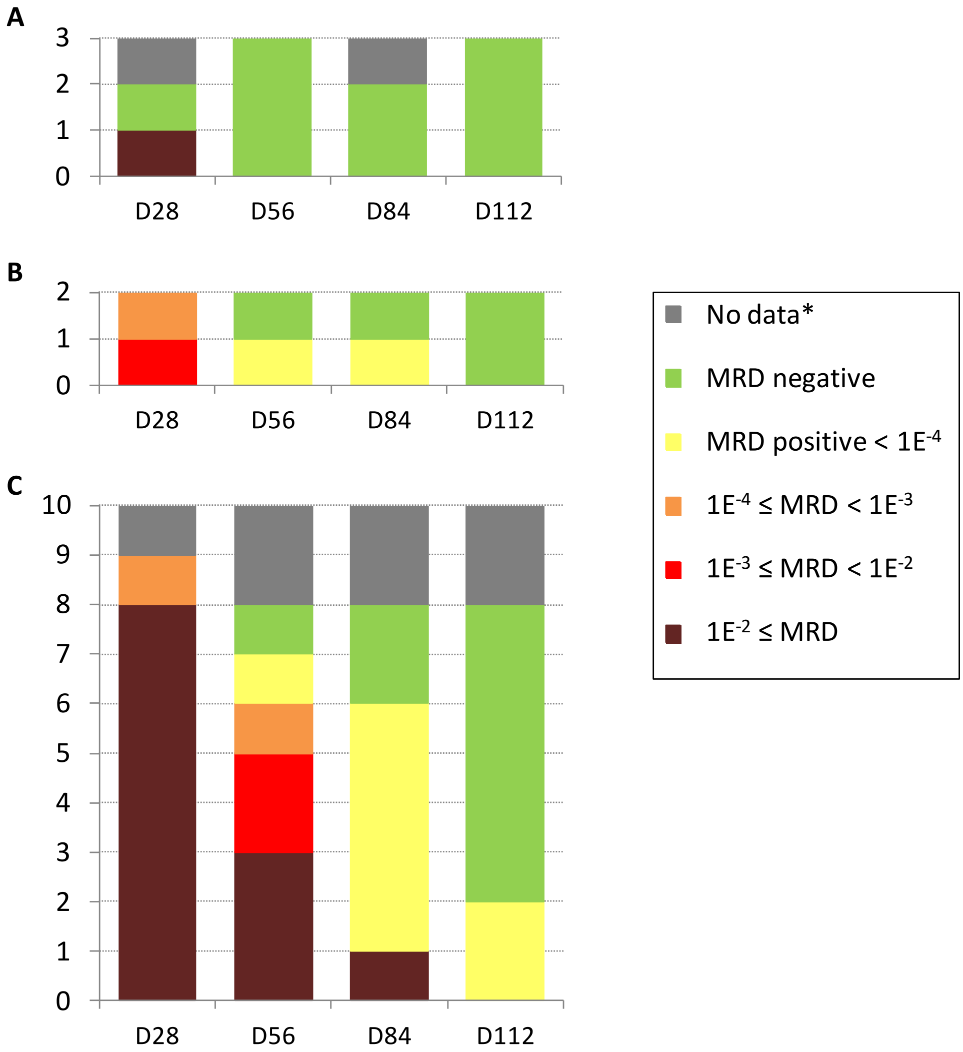

The figure shows MRD clearance in 15 patients with *PML::RARA*-positive AML (APL). Three patients were treated according to the AML-BFM 2012 registry protocol. Their treatment consisted of 4 blocks of chemotherapy and ATRA (Supplemental figure 1) (A). Two patients were treated according to the high risk arm of the amended AML-BFM 2012 registry protocol, they received 1 induction block of chemotherapy, ATRA and ATO (B). Eight and two patients were treated according to the standard risk arm of the amended AML-BFM 2012 protocol and the ICC APL study 02 protocol, respectively, their treatment consisted of ATO and ATRA blocks without chemotherapy (C). One of the two patients treated according to the ICC APL study 02 protocol had pre-treatment white blood cell count ≥ 10 x 10^9 / L and received gemtuzumab ozogamicin.

D - day; * BM sample was not available or the time point was not reached.

Supplemental Figure 5. MRD levels in paired bone marrow - peripheral blood samples

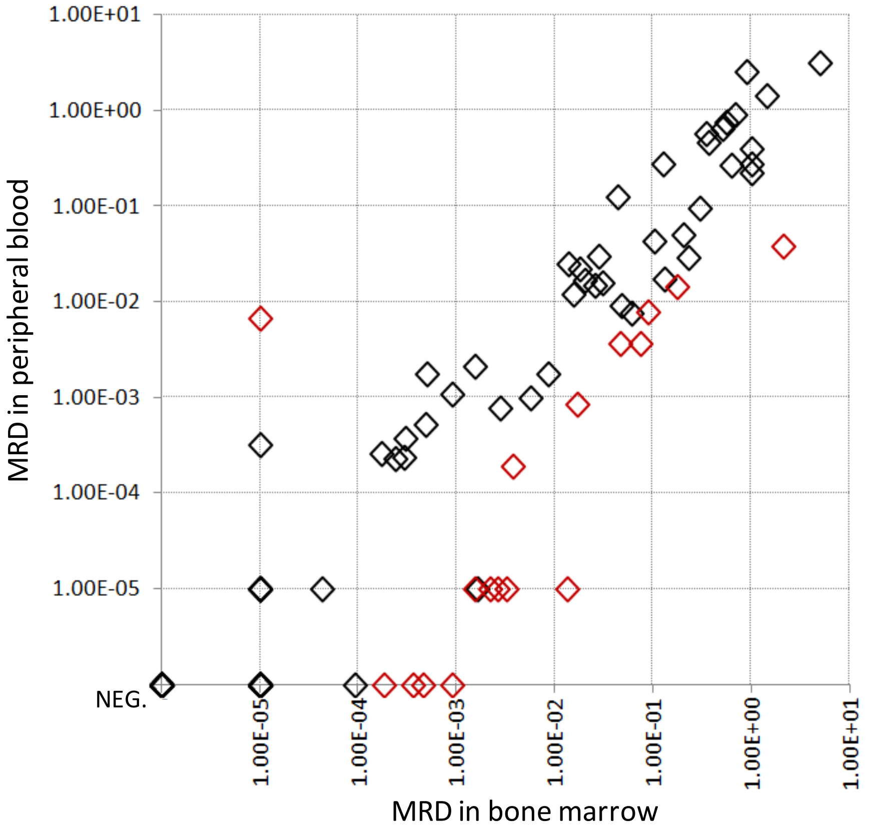

Figure shows DNA-based MRD levels in 105 paired samples collected at different time points during and after treatment in 27 patients (1-14 samples per patient, median = 3 samples per patient) with 8 different AML subtypes (1-6 patients per subtype). For graphical representation, non-quantifiable positive samples were assigned an MRD value of 1.00E-05. Paired samples were considered concordant if A) the difference in quantifiably positive MRD levels in peripheral blood (PB) and bone marrow (BM) was ≤1 log, B) the level in one compartment was quantifiably positive at ≤1 log above the quantitative range/sensitivity, while the MRD in the other compartment was non-quantifiably positive/negative. The remaining samples were considered discordant. Concordant samples (n=88) are shown in black, discordant samples (n=17) in red. For all discordant samples except one, MRD was higher in BM than in PB. Discordant samples were found in 10/27 patients: in 4/6 patients with *CBFB::MYH11*, 2/4 patients with *RUNX1::RUNX1T1*, 1/6 patients with *PML::RARA*, 0/3 patients with *CEBPA*m, 1/2 patients with *NPM1*m, 1/1 patient with *WT1*m, 1/1 patients with FLT3-ITD, 0/1 patients with *DEK::NUP214*, 0/1 patients with *ETV6::CTNNB1*, 0/1 patients with *KMT2A::MLLT10* and 0/1 patients with *KMT2A::ABI1*.

Supplemental Figure 6. Outcome of patients stratified by treatment arm and (cyto)genetic risk

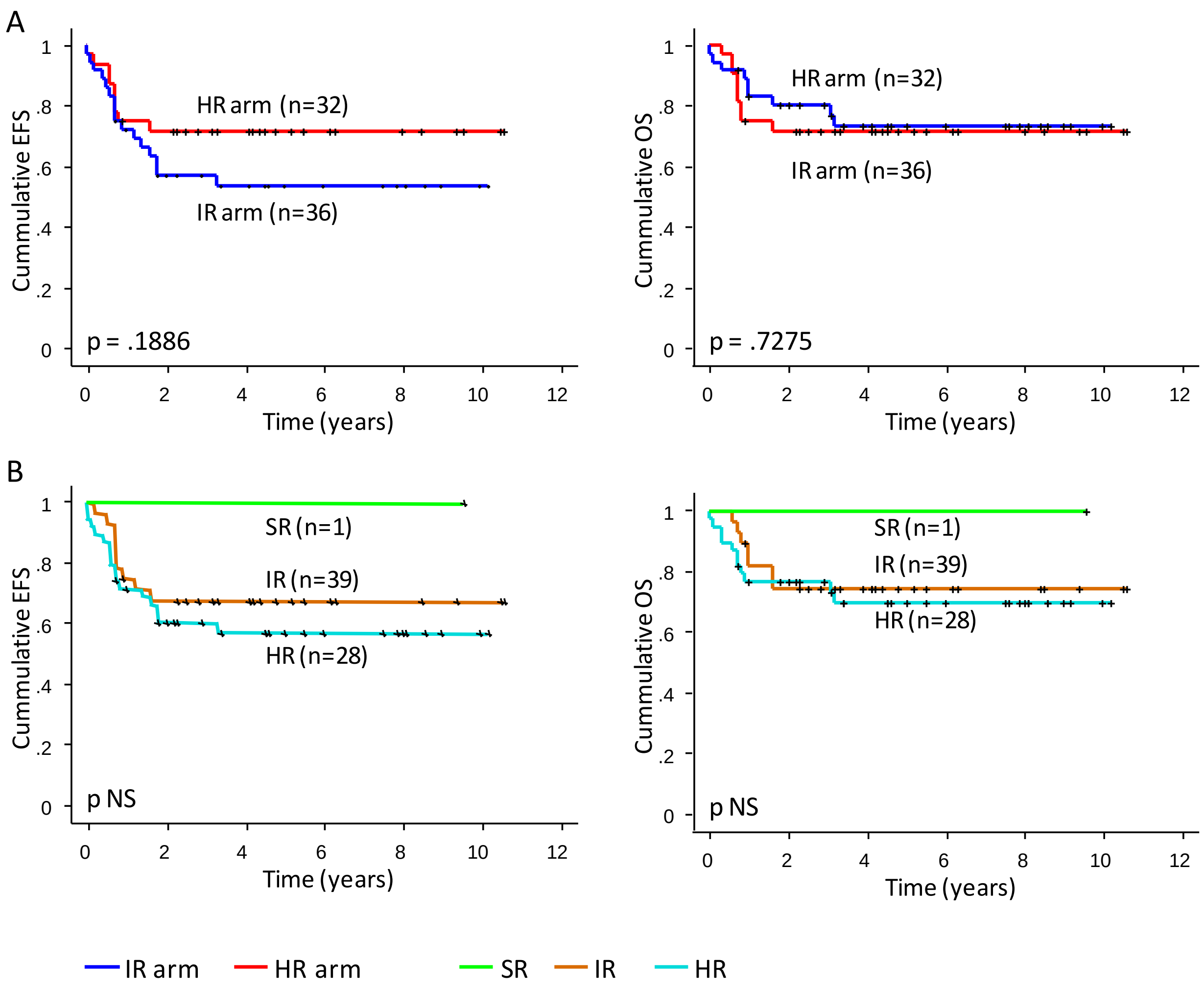

The figure shows treatment outcomes for 68 patients treated in the IR and HR arms of the 2012 BFM AML Registry protocol stratified by treatment arm (A) or (cyto)genetic risk (B). Treatment on HR arm included hematopoietic stem cell transplantation, treatment on IR arm comprised of chemotherapy only. EFS – event free survival, OS – overall survival. Censoring is indicated by crosses.
